# Efficacy of an Electronic Health Management Program for Patients With Cardiovascular Risk: Randomized Controlled Trial

**DOI:** 10.2196/15057

**Published:** 2020-01-22

**Authors:** Young Ho Yun, EunKyo Kang, Young Min Cho, Sang Min Park, Yong-Jin Kim, Hae-Young Lee, Kyae Hyung Kim, Kiheon Lee, Hye Yeon Koo, Soojeong Kim, YeEun Rhee, Jihye Lee, Jeong Hee Min, Jin-Ah Sim

**Affiliations:** 1 Seoul National University College of Medicine Department of Family Medicine Seoul Republic of Korea; 2 Seoul National University College of Medicine Department of Internal Medicine Seoul Republic of Korea; 3 Bundang Seoul National University Hospital Department of Family Medicine Seongnam Republic of Korea; 4 Seoul National University College of Medicine Cancer Research Institute Seoul Republic of Korea

**Keywords:** health, hypertension, diabetes, hypercholesterolemia, randomized controlled trial

## Abstract

**Background:**

In addition to medication, health behavior management is crucial in patients with multiple risks of cardiovascular mortality.

**Objective:**

This study aimed to examine the efficacy of a 3-month Smart Management Strategy for Health–based electronic program (Smart Healthing).

**Methods:**

A 2-arm randomized controlled trial was conducted to assess the efficacy of Smart Healthing in 106 patients with at least one indicator of poor disease control and who had hypertension, diabetes, or hypercholesterolemia. The intervention group (n=53) took part in the electronic program, which was available in the form of a mobile app and a Web-based PC application. The program covered 4 areas: self-assessment, self-planning, self-learning, and self-monitoring by automatic feedback. The control group (n=53) received basic educational material concerning disease control. The primary outcome was the percentage of participants who achieved their clinical indicator goal after 12 weeks into the program: glycated hemoglobin (HbA_1c_) <7.0%, systolic blood pressure (SBP) <140 mmHg, or low-density lipoprotein cholesterol <130 mg/dL.

**Results:**

The intervention group showed a significantly higher success rate (in comparison with the control group) for achieving each of 3 clinical indicators at the targeted goal levels (*P*<.05). Only the patients with hypertension showed a significant improvement in SBP from the baseline as compared with the control group (72.7% vs 35.7%; *P*<.05). There was a significant reduction in HbA_1c_ in the intervention group compared with the control group (difference=0.54%; *P*≤.05). In the intervention group, 20% of patients with diabetes exhibited a ≥1% decrease in HbA_1c_ (vs 0% among controls; *P*≤.05).

**Conclusions:**

A short-term self-management strategy-based electronic program intervention may improve clinical outcomes among patients with cardiovascular risks.

**Trial Registration:**

ClinicalTrials.gov NCT03294044; https://clinicaltrials.gov/ct2/show/NCT03294044

## Introduction

### Background

Hypertension, diabetes, and hypercholesterolemia are the global leading risks of cardiovascular mortality [1-3]. Health behaviors such as engaging in exercise, balanced diet, and weight control reduce one’s risk of cardiovascular mortality. Therefore, in addition to medication, the management of health behavior is crucial in patients with multiple risks of cardiovascular mortality [4]. Clinical guidelines recommend a combined self-management strategy of health behaviors and appropriate medication use [3,5] A recent self-management approach in line with the Chronic Care Model (CCM) specifies that health behavior management should be used to manage coexisting illnesses [6-8]. Owing to the importance of self-management in patient-centered health care in combination with the increased use of mobile devices (including smartphones and tablets), there is a need to develop an efficient, affordable, and sustainable self-management strategy-based elecronig program that targets high-risk individuals [9-11].

Research concerning mobile health (mHealth) innovations to support populations with chronic illnesses and improve their health behaviors is growing [4,9,11]. A systematic review showed that the use of apps in mHealth has the potential to improve health outcomes among patients with chronic diseases through enhanced self-management [12]. A number of randomized controlled trials (RCTs) have assessed the effectiveness of mobile phone- or tablet-assisted self-management programs in addressing cardiovascular disease [13] or chronic hepatitis [14]. Although there is a need to organize intervention programs to improve the health outcomes of patients with chronic illnesses [6], few mHealth trials have addressed this [3,5].

### Objectives

We therefore aimed to determine the efficacy of a self-management strategy-based electronic program for patients who had been treated for hypertension, diabetes, or hypercholesterolemia and who had at least one indicator of poor disease control. To do so, we provided patients with an intervention program via a Web-based health management program (mobile app or PC-Web-based) [15].

## Methods

### Study Design

**Figure 1:**
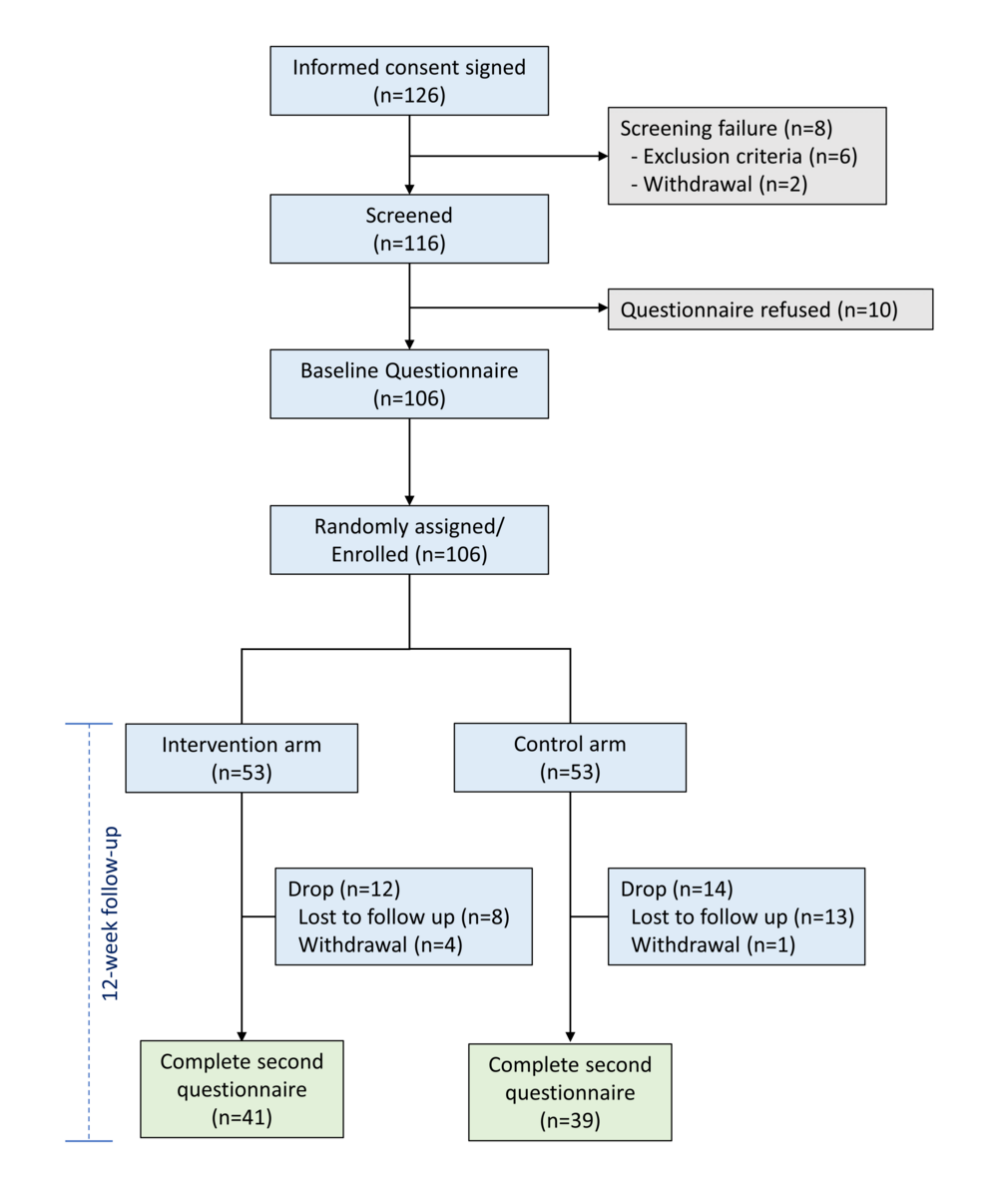
Flowchart depicting the study methodology

We conducted this study with 106 patients within 2 months of treatment termination, and the patients were randomly assigned to either the control group or the intervention group (ie, *Smart Healthing*; Multimedia Appendices 1 and 2). Each physician from 2 study hospitals screened patients for the eligibility criteria by reviewing their medical records and blood test results at outpatient clinics. A clinical research coordinator at each hospital explained the study details to the participants who met the eligibility criteria (Figure 1). The patients who were eligible to participate were recruited by the physician in-charge and were asked to provide written informed consent to the researchers. The institutional review boards at the 2 hospitals approved the study protocol (numbers 1707-084-870 and B-1802/453-401). The trial was performed in accordance with the Good Clinical Practice guidelines and the Declaration of Helsinki. All staff who were involved in screening and recruiting participants were certified by their institutions for ethical conduct of research (Collaborative Institutional Training Initiatives).

### Participants

From November 2017 to March 2018, we identified patients with at least one indicator of poor disease control among patients who had been treated for hypertension, diabetes, or hypercholesterolemia. We recruited patients who met the following criteria: (1) aged ≥19 years; (2) diagnosed with hypertension, diabetes, or hypercholesterolemia; (3) failed to meet 1 or more of the following clinical goals: (i) glycated hemoglobin (HbA_1c_) <7.0%, (ii) systolic blood pressure (SBP) <140 mmHg, or (iii) low-density lipoprotein (LDL) cholesterol <130 mg/dL; (4) had a smartphone and personal computer (for the electronic program-based health care program); and (5) understood the study’s purpose.

Patients were excluded from the study if they met any of the following criteria: (1) had medical conditions that would limit participation adherence (as confirmed by their referring physician [eg, dyspnea and severe depression]); (2) could not speak, understand, or write Korean; or (3) could not understand the content of the provided materials owing to poor eyesight and/or hearing.

### Randomization

We used an internet-based Clinical Research and Trial management system by the Centers for Disease Control and Prevention for participant randomization. The patients were randomly assigned (1:1) to the intervention or control group based on a random computer-generated number. To minimize the effects of potential confounding variables, we randomized participants stratified by disease type with the clinical indicators (hypertension, diabetes, or hypercholesterolemia). The research assistants executed face-to-face procedures and therefore could not be blinded when assigning participants to groups.

### Control

The attention control group was encouraged to continue their usual care and routine medications and to study a health educational booklet about chronic diseases. The booklet noted 12 healthy life habits: positive thinking, regular exercise, balanced diet, proactive living, regular checks-ups, helping others, regular religious life, quitting smoking, drinking cessation, work-life balance, living with loved ones, and taking medication.

### Intervention

The intervention group received the self-management strategy-based electronic program, whereas the control group received basic educational material about disease content. We developed the Smart Management Strategy for Health–based electronic program and utilized the comprehensive and multifaceted Smart Management Strategy for Health strategies. The self-management strategy-based electronic program used in this study was a 3-month Smart Management Strategy for Health Intervention, and it is comprised of an app and a Web-based program. On the basis of our literature review and interviews, we developed a conceptual framework for the Smart Management Strategy for Health intervention that incorporates management strategies for overcoming crises and developing healthy management strategies. The Smart Management Strategy for Health intervention includes the following 9 strategies: (1) assessment, (2) reality acceptance, (3) preparation for change, (4) decision making, (5) planning, (6) environment creation, (7) action, (8) feedback and maintenance, and (9) core strategies. All of these strategies can help patients overcome a disease crisis and develop healthy self-management skills [16,17].

The program covered 4 areas: self-assessment, self-planning, self-learning, and self-monitoring by automatic feedback. We targeted 4 priority areas for intervention—positive thinking, balanced diet, physical activity, and medication. The 20 learning sessions included *12 Rules for Highly Effective Health Behavior* and health management strategies.

The self-management strategy-based electronic program was used for 12 weeks. The patients were provided with a manual with detailed instructions on how to use the program to both increase its usage rate and decrease the dropout rate. Self-evaluations were conducted with regard to the participants’ self-management competence and health practices before and after the program (excellent, moderate, and poor). In addition, the patients wrote health mission statements that included their life goals, health practice goals, obstacles, and methods to overcome them and detailed promises in relation to the self-management strategy-based electronic program. Self-learning was structured with the health management strategy and health information on 12 health behavior rules. The patients received daily health educational content from the self-management strategy-based electronic program. Every week, the patients learned 1 health behavior among the 4 essential rules, and they could selectively study the other 8 health behavior rules. By graphically displaying the participants’ blood glucose levels, blood pressure, and weight to them, it was possible for the participants to track any changes.

The patients could create their own health management weekly plan for the 4 essential health behavior rules and monitor their progress and health. The weekly plan addressed dieting, vegetable and fruit consumption, physical activity, and daily medication schedule. More specifically, the weekly physical activity plan included the activity’s type, length of time, intensity, and schedule. The self-management strategy-based electronic program included an automatic push function and alarms for the scheduled physical activities, medications, and assessments to remind participants of their plans. After 1 week, the patients were provided with feedback to motivate and help them plan for the following week. Through periodic monthly assessments, the program identified changes in their essential health behaviors and provided feedback on monthly changes through a comparison of their prior month’s results to help patients change their behavior.

### Measures

The primary outcome was the percentage of subjects that met the target clinical indicators (HbA_1c_ <7.0%, SB*P*<140 mmHg in clinic, or LDL cholesterol <130 mg/dL).

The secondary outcomes included the originally proposed clinical indicator outcomes—physical activity, depression, self-management strategies, and health behaviors after 12 weeks in the program. The patients’ self-management strategies were assessed with a short form of the Smart Management Strategy for Health, which is a 3-set, 16-factor, 30-item tool (ie, core strategies, 10 items; preparation strategies, 10 items; and implementation strategies, 10 items) that assesses patients’ abilities to overcome health-related crises [17]. Physical activity was measured with the modified version of the Godin Leisure-time Exercise Questionnaire, which is widely used, reliable, and valid [16]. The modified version adds average duration to the original questions of average frequency of light, moderate, and strenuous exercise per week. We evaluated depression with the Patient Heath Questionnaire-9 (PHQ-9). The participants were asked to measure their 12 health behaviors with 5 scales: (1) precontemplation, (2) contemplation, (3) preparation, (4) action, and (5) maintenance, which are all based on the transtheoretical model [18,19].

We assessed the proportion of patients with a ≥1.0% decrease in their HbA_1c_ level from the baseline, the proportion of patients with a ≥10-mmHg decrease in SBP from the baseline, and a ≥15% decrease in LDL cholesterol level. We also assessed the proportion of patients with either a decrease or no change in PHQ-9 score from the baseline, a ≥5- metabolic equivalent of task increase in physical activity level, a ≥10% increase in self-management strategy, and a ≥3 habits increase in the maintenance of the 12 health habits.

The participants completed baseline questionnaires before randomization at the clinics. After 12 weeks, we conducted follow-up assessments with the participants with regard to the primary and secondary outcomes. When patients did not complete a questionnaire item, the clinical research coordinator documented the reason.

### Statistical Analysis

Providing 80% power to detect a 30% proportion difference in patients achieving disease control with a 2-tailed alpha value of less than .05, we calculated that it was necessary to have 42 patients per group. We predicted a 20% dropout rate and aimed at recruiting 53 patients in each group. A multiple imputation approach was used to impute scores for missing values for the intent-to-treat analysis. The imputed values were used for the covariates analyses but not for the descriptive statistics.

We used a Student *t* test or Pearson chi-square test to determine significant differences in the baseline characteristics between the intervention and control groups. We used an analysis of covariance to estimate between-group changes in the clinical outcome numbers with general linear modeling, adjusting for the baseline score and age. We compared the participants’ changes from their baseline values with their values after 12 weeks in the program. Pearson chi-square test was used to assess between-group differences in the proportion of patients with improvement (overall, depression, and physical activity). Enhanced self-management strategies and health habits were also estimated by using a Pearson chi-square test.

We used STATA version 14.2 (STATA) for all statistical analyses. A two-sided *P* value <.05 was considered significant.

## Results

### Study Participants

The study team contacted 281 patients between October 27, 2017, and March 26, 2018. Of these, 124 patients were eligible, and 18 were excluded because of screening failures or refusal to participate. Finally, 53 were randomized to the intervention group and 53 to the control group (Figure 1). Except for age and residence, all baseline characteristics were similar between the 2 groups (Table 1). More specifically, compared with the control group, the intervention group was older, and they were more likely to reside in metropolitan areas (*P*=.001 and .04, respectively).

**Table 1:** Baseline characteristics of participants.

Characteristics			Intervention group (n=53), n (%)	Control group (n=53), n (%)	
**Age (years)**					
	20-49		8 (13)	18 (34)	
	50-59		17 (33)	21 (40)	
	60-69		26 (50)	8 (15)	
	≥70		2 (4)	6 (11)	
**Sex**					
	Male		31 (58)	29 (55)	
	Female		22 (42)	24 (45)	
**Marital status**					
	Married		47 (89)	45 (85)	
	Unmarried		4 (8)	6 (11)	
	Separated/bereaved		2 (4)	2 (4)	
**Educational status**					
	High school or less		18 (34)	18 (34)	
	≥College or university		35 (66)	35 (66)	
**Presence of religion**					
	Yes		36 (68)	27 (51)	
	No		17 (32)	26 (49)	
**Residence**					
	Metropolitan		39 (74)	28 (52)	
	Urban or rural		14 (26)	25 (47)	
**Monthly income (1000 KRW^a^/month)**					
	≤3999		14 (26)	22 (42)	
	4000-4999		10 (19)	9 (17)	
	≥5000		29 (55)	22 (42)	
**Employment status**					
	Employed		36 (68)	33 (62)	
	Unemployed/retired		17 (32)	20 (38)	
**Disease^b^**					
	Diabetes mellitus		26 (49)	21 (40)	
	Dyslipidemia		23 (43)	23 (43)	
	Hypertension		11 (21)	14 (26)	

^a^KRW: Korean Won.

^b^Some participants have been diagnosed with more than one disease.

### Success Rate for Achieving Goals

Table 2 describes the percentage of patients’ achieved goals. The intervention group showed a significantly higher success rate for achieving the targeted levels for each of the 3 clinical indicators after 12 weeks, and this higher success rate remained significant when stratified by starting medication with the Mantel-Haenszel method (*P*<.05). With regard to disease, the patients with hypertension in the intervention group showed significant improvement compared with the control group (72.7% vs 35.7%, *P*=.035; Mantel-Haenszel chi-square test). These results for the patients with diabetes and hypercholesterolemia were nonsignificant.

We found a significant reduction of HbA_1c_ in the intervention group compared with the control group (0.71 vs 0.22, respectively; between-group difference=–0.54, 95% CI –0.98 to –0.11; *P*=.014). The patients with hypertension exhibited a greater reduction in SBP in the intervention group compared with the control group; however, this result was nonsignificant (17.5 mmHg vs 11.6 mmHg; *P*=.41). For the patients with hypercholesterolemia, both the intervention and control groups showed a reduction in LDL cholesterol, and the between-group difference was nonsignificant (23.7 mg/dL vs 25.3 mg/dL; *P*=.72).

**Table 2:** Differences in clinical outcomes controlling for the primary disease.

Time point		Intervention group (n=60)			Control group (n=57)			*P* value^a^		*P* value^b^
		Success, n	Change (%)	Success, n		Change (%)				
**All diseases**			60			37	.01		.02	
	Baseline	0		0						
	12 weeks	36		21						
**Diabetes^c^**			54			38	.35		.43	
	Baseline	0		0						
	12 weeks	14		8						
**Hypertension^d^**			73			36	.07		.04	
	Baseline	0		0						
	12 weeks	8		5						
**Dyslipidemia^e^**			61			35	.08		.1	
	Baseline	0		0						
	12 weeks	14		8						

^a^All reported *P* values are 2-sided, with *P*<.05 considered as statistically significant.

^b^Stratified analysis by starting medication (Mantel-Haenszel method).

^c^Intervention: n=26; control: n=21.

^d^Intervention: n=11; control: n=14.

^e^For both intervention and control: n=23.

### Differences in Other Clinical Outcomes, Health Outcomes, and Self-Management Measures

Among the intervention group, 20% of patients with diabetes exhibited a ≥1% decrease in HbA_1c_ (compared with 0% in the control group; Table 3).

In the intervention group, 73% of the participants showed a decrease or no change in depressive symptoms (vs 51% in the control group). The Smart Healthing program strengthened the implementation strategy of the modified Smart Management Strategy for Health greater in the intervention group (57.5%) than in the control group (33.3%). However, the differences in the core and preparation strategies for both the intervention and control groups were nonsignificant (*P*=.53 and .30, respectively; Table 4). There were no important harms or unintended effects observed in either group.

**Table 3:** Differences in clinical measures.

Differences (clinical outcomes)	Intervention group (n=53), n (%)	Control group (n=53), n (%)	*P* value
≥1.0 percentage point decrease in glycated hemoglobin level (intervention: n=25; control n=19)	5 (20)	0 (0)	.04
≥10 mmHg decrease in systolic blood pressure (intervention: n=10; control: n=9)	8 (80)	5 (56)	.25
≥15% low-density lipoprotein decrease (intervention: n=15; control: n=17)	7 (47)	7 (41)	.76

**Table 4:** Differences in health outcomes and self-management measures.

Differences			Intervention group (n=53), n (%)		Control group (n=53), n (%)			*P* value
**Health outcomes**								
	Decrease or no change in PHQ-9^a^ score (intervention: n=41; control: n=39)	30 (73)		20 (51)			.04	
	≥5 metabolic equivalent of task physical activity (intervention: n=41; control: n=39)	29 (71)		32 (82)			.23	
	≥Increase in 3 of the 12 health habits (intervention: n=41; control: n=39)	12 (29)		11 (28)			.92	
**Self-management strategies**								
	≥10% increase in the *Core Strategy of SAT*^b^ (intervention: n=41; control: n=39)	13 (32)		15 (38)			.53	
	≥10% increase in the *Preparation Strategy of SAT* (intervention: n=38; control: n=39)	15 (39)		20 (51)			.30	
	≥10% increase in the *Implementation Strategy of SAT* (intervention: n=40; control: n=33)	23 (58)		13 (33)			.03	

^a^PHQ-9: Patient Health Questionnaire-9.

^b^SAT: Smart Management Strategy for Health Assessment Tool.

## Discussion

### Principal Findings

This RCT indicated that this study’s self-management strategy-based electronic program effectively encouraged patients with at least one indicator of poor disease control for diabetes, hypertension, or hypercholesterolemia to meet key guideline criteria (HbA_1c_, SBP, and LDL cholesterol). The patients with hypertension showed a significant improvement in SBP from their baseline values in comparison with the control groups. There was also a significant reduction in HbA_1c_ in the intervention group compared with the control group. We are particularly encouraged by these findings, and we posit that this study’s self-management strategy-based electronic program can more effectively support disease control in comparison with the usual care strategies.

The proportion of patients with controlled hypertension increased significantly more in the intervention group than in the control group. The proportion of patients with controlled diabetes and hypercholesterolemia also increased more in the intervention group than in the control group; however, these findings were nonsignificant, which could be a result of the study’s small sample size. These improvements in the primary outcomes in our trial support the findings from earlier trials with same clinical indicators. Concerning the secondary outcomes, the mean change in HbA_1c_ and the proportion of patients with a significant decrease in HbA_1c_ level from their baseline values were both higher in the intervention than the control (ie, usual care) group [3,6,20-24].

There are several possible explanations for our findings. First, the intervention strategies were based on the Smart Management Strategy for Health program. The intervention significantly increased the participants’ Implementation Strategy scores for self-management. It is possible that the CCM self-management program thus helps individuals to develop preferences for how to manage their own care [7,8] It is assumed that most patients want to remain independent; however, these preferences and patients’ daily behaviors may change over time because of their symptoms, the treatments they undergo and their goals [8]. Patients with chronic illnesses must manage the medical and emotional strain of their health condition(s) [8,25]. The Smart Management Strategy for Health supports patients to help them overcome a disease crisis and develop health-related self-management skills [26,27] The fact that this intervention integrates self-management strategies with electronic program in the CCM highlights how mHealth can address cardiovascular risks [28].

Second, a user‐centered electronic program has the potential to improve clinical indicators among those living with chronic diseases by allowing users to obtain information from the mobile- and Web-based pages at their own pace, to flexibly review material as needed [29,30] and by facilitating the management of multiple health behaviors [11]. Third, the noted self-management program can help patients by providing immediate, easy, and continual access to the intervention [4,8,15]. This electronic program intervention may thus provide a critical route to successful chronic care. From a clinical perspective, it could be valuable to link this electronic program-based program with face-to-face or telephone counseling [31-33].

Furthermore, we did not observe any significant changes concerning the examined health habits. There are 2 possible explanations for this finding. First, the intervention might not have been intensive enough to modify patients’ long-held health habits. Second, the 12-week intervention period might have been too short to observe any meaningful changes in health habits [5].

Despite well-established data on chronic disease management, its uptake into routine clinical practice remains limited. Further innovation, optimization, and rigorous research in customized mobile technology might improve health care delivery and outcomes [5,12].

### Limitations

Several limitations of our study should be noted. First, our sample included 3 types of cardiovascular risks of varying severity and a small number of patients; thus, we lacked the power to determine meaningful differences. Further studies with larger sample sizes and distinct cardiovascular risks are needed to confirm the efficacy of the intervention. Second, approximately one-quarter of the patients in the intervention group did not complete the follow-up at the 12-week mark. Missing data for these patients may have resulted in an underestimation of the efficacy of the intervention program. Third, as the patients were aware of their group, the self-reported changes in depression, physical activity, self-management strategy, and health behaviors could have been influenced by that awareness and not just by the intervention itself. Fourth, there might be attrition bias. A quarter of the intervention group was lost to follow up, and many patients of this group were older, which may be associated with less use of newer technology. This loss may lead to an overestimate of effect. Fifth, although the attention control group was encouraged to continue their usual care and routine medications and to study a health educational booklet about chronic diseases, the Hawthorne effect may be still relevant. Finally, our trial was relatively short, and we do not know whether the changes associated with the program would be maintained over a longer period. Additional research on the long-term efficacy of this intervention, including a full-scale RCT, is warranted to confirm the efficacy of this program.

### Conclusions

A short-term self-management strategy-based electronic program intervention may improve clinical outcomes among patients with cardiovascular risks. More research with context-specific trials is needed to enhance these findings, to ensure the long-term generalizability and sustainability of the program, and to indicate the cost-effectiveness of this intervention.

## Appendix

Multimedia Appendix 1 Screenshot of the app.

Multimedia Appendix 2 Screenshot of the intervention (Smart Healthing).

Multimedia Appendix 3 CONSORT-EHEALTH checklist (V 1.6.1).

## Abbreviations

CCM: chronic care model
HbA_1c_: glycated hemoglobin
LDL: low-density lipoprotein
mHealth: mobile health
PHQ-9: Patient Health Questionnaire-9
RCT: randomized controlled trial
SBP: systolic blood pressure
