# Supplementary figures and images for: Efficacy of an Electronic Health Management Program for Patients With Cardiovascular Risk: Randomized Controlled Trial
Source: J Med Internet Res. 2020 Jan 22;22(1):e15057. doi: 10.2196/15057 (PMC7003122; doi:10.2196/15057)

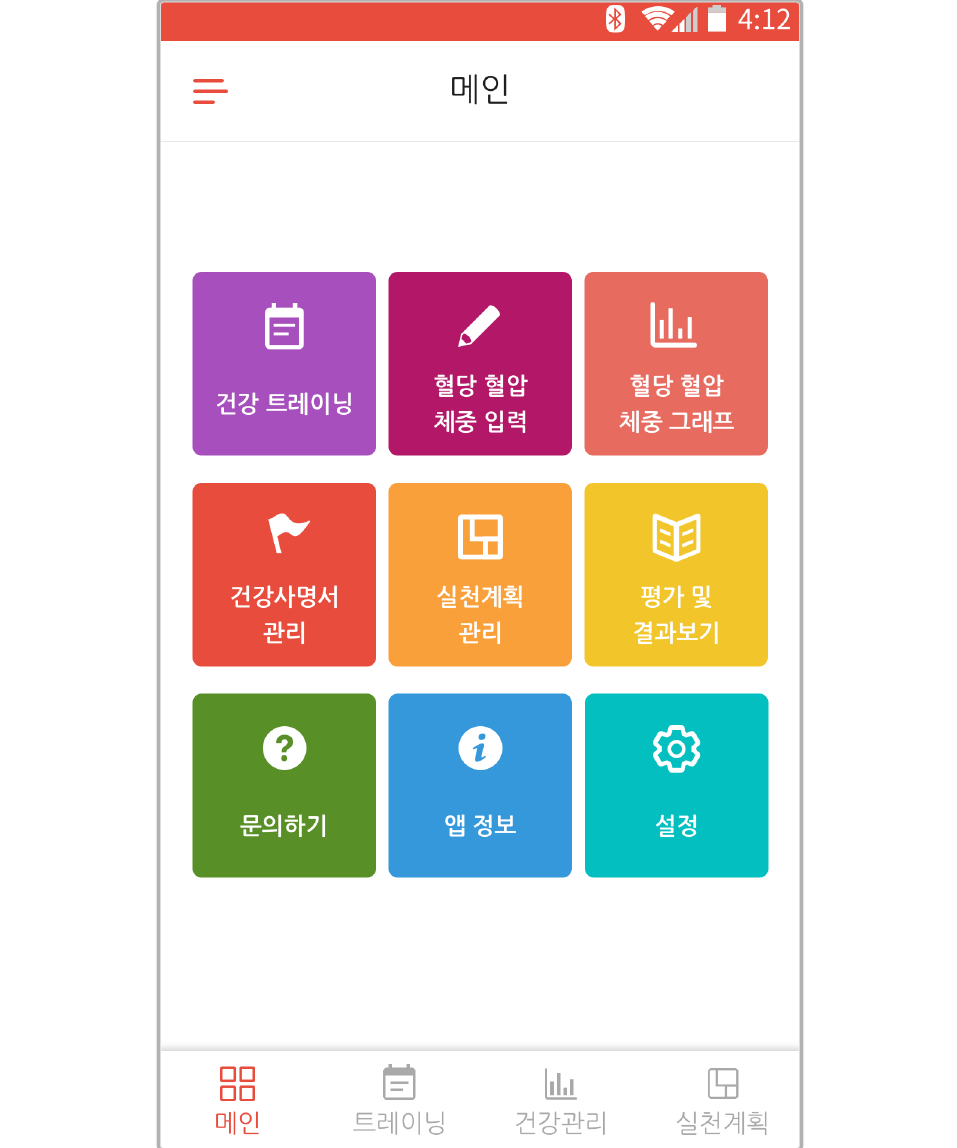

Supplement: Multimedia Appendix 1 [file jmir_v22i1e15057_app1.png]

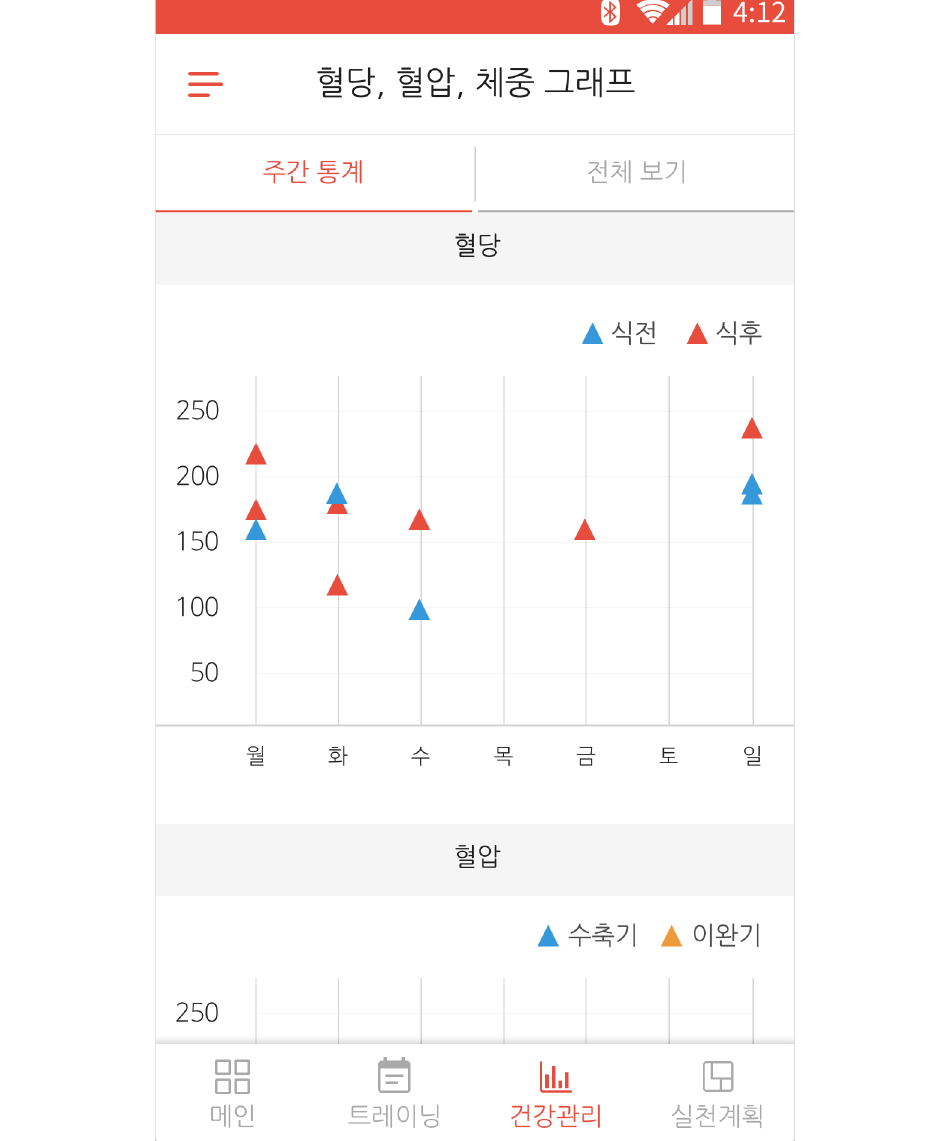

Supplement: Multimedia Appendix 2 [file jmir_v22i1e15057_app2.png]
